# Obesogenic and Ketogenic Diets Distinctly Regulate the SARS-CoV-2 Entry Proteins ACE2 and TMPRSS2 and the Renin-Angiotensin System in Rat Lung and Heart Tissues

**DOI:** 10.3390/nu13103357

**Published:** 2021-09-25

**Authors:** Daniel Da Eira, Shailee Jani, Rolando B. Ceddia

**Affiliations:** Muscle Health Research Center, School of Kinesiology and Health Science, York University, Toronto, ON M3J1P3, Canada; DanielDaEira@hotmail.com (D.D.E.); janishailee@gmail.com (S.J.)

**Keywords:** obesity, ketones, inflammation, TLR4, IL6, TNF-alpha, MasR

## Abstract

Background: Obesity increases the severity of SARS-CoV-2 outcomes. Thus, this study tested whether obesogenic and ketogenic diets distinctly affect SARS-CoV-2 entry proteins and the renin-angiotensin system (RAS) in rat pulmonary and cardiac tissues. Methods: Male Sprague-Dawley rats were fed either standard chow (SC), a high-fat sucrose-enriched diet (HFS), or a ketogenic diet (KD) for 16 weeks. Afterwards, levels of angiotensin converting enzyme 2 (ACE2), transmembrane protease serine 2 (TMPRSS2), RAS components, and inflammatory genes were measured in the lungs and hearts of these animals. Results: In the lungs, HFS elevated ACE2 and TMPRSS2 levels relative to SC diet, whereas the KD lowered the levels of these proteins and the gene expressions of toll-like receptor 4 and interleukin-6 receptor relative to HFS. The diets did not alter ACE2 and TMPRSS2 in the heart, although ACE2 was more abundant in heart than lung tissues. Conclusion: Diet-induced obesity increased the levels of viral entry proteins in the lungs, providing a mechanism whereby SARS-CoV-2 infectivity can be enhanced in obese individuals. Conversely, by maintaining low levels of ACE2 and TMPRSS2 and by exerting an anti-inflammatory effect, the KD can potentially attenuate the severity of infection and migration of SARS-CoV-2 to other ACE2-expressing tissues.

## 1. Introduction

Obesity is characterized by an expansion in white adipose tissue (WAT) mass and is linked to a number of comorbidities, including hypertension and diabetes [1]. More recently, obesity has emerged as a major risk factor for severe illness related to COVID-19 infection [2,3]. Infection with the severe acute respiratory syndrome-coronavirus-2 (SARS-CoV-2) occurs mainly through the airway [4]. At the cellular level, the virus binds to its receptor, ACE2 [4,5]. The interaction between ACE2 and SARS-CoV-2 occurs via the virus’s spike (S) protein; however, an additional crucial step, catalysed by transmembrane protease serine 2 (TMPRSS2), leads to cleavage of the S protein [4,5]. This latter step allows for the fusion of the virus and host cell membranes, so endocytosis of the virus can occur. Once inside the cell, the virus can utilize the cell’s ribosomes to transcribe its own RNA and undergo replication [6]. The enhanced susceptibility of obese individuals to severe COVID-19 outcomes has been linked to elevations in ACE2 and TMPRSS2 in lung tissues [4,7]. In fact, Sarver and Wong showed that ACE2 expression was elevated in the lungs and tracheas of male, obese mice [4]. Similarly, Batchu et al. reported elevations in ACE2 and TMPRSS2 protein contents in the lungs of HF-fed, diabetic mice [7]. In this context, the diet-induced elevations in SARS-CoV-2 entry proteins provide a plausible mechanism that explains the increased susceptibility of obese individuals to severe outcomes. It is important to note that ACE2 is also a component of the renin-angiotensin aldosterone system (RAS), which has emerged as another possible mechanism mediating COVID-19-related illness [8].

The RAS is classically responsible for the regulation of fluid balance and blood pressure [8]. In short, the kidney releases renin, which converts liver-derived angiotensinogen into angiotensin I (Ang-I) [9]. Ang-I is then processed into angiotensin-II (Ang-II), mainly in the pulmonary circulation [10], by the angiotensin converting enzyme (ACE1) [9]. Ang-II can then bind to the G-protein coupled receptor angiotensin-II, type 1 receptor (AT_1_R) [9] where it induces vasoconstriction, water retention and sympathetic nervous system activation [9]. Under conditions of hypertension and obesity, hyperactivation of the RAS leads to inflammation, fibrosis and oxidative damage [8]. In this context, therapies aimed at increasing flux through the counterregulatory arm of the RAS have gained attention as a potential treatment for hypertension and obesity [2,8]. These effects are mainly mediated by ACE2, which converts Ang-II into Ang(1,7) [8]. Ang(1,7) binds to the Mas receptor (MasR), which exerts protective, anti-inflammatory effects [9]. Moreover, Ang-II can also bind to angiotensin II, type 2 receptor (AT_2_R) [2]. AT_2_R exerts similar effects to MasR, however it is mainly expressed under conditions of AT_1_R hyperactivation and injury to suppress inflammation and oxidative stress [2,11]. Following COVID infection, ACE2 becomes downregulated [2]. Consequently, the obesity-induced elevation in Ang-II increases signalling through AT_1_R, which promotes inflammation and tissue damage [2]. Thus, the role of ACE2 in COVID-19-related illness extends beyond serving as a viral entry protein. Rather, it is also implicated in the inflammatory response that ensues, which carries a profound impact for tissue damage and organ failure [2]. In this context, shifting obese individuals from the ACE1/Ang-II/AT_1_R arm to the ACE2/Ang(1,7)/MasR and AT_2_R arm is of great importance to reduce the susceptibility of obese individuals to severe COVID-19 outcomes.

Recently, the ketogenic diet (KD) has sparked interest as a potential therapeutic tool for the management of obesity. In fact, it has been shown that the KD improved blood pressure, glycemia and body weight in Type 2 diabetic patients [12] and carries potential as an anti-inflammatory diet [13]. However, to our knowledge, little is known regarding the effect of the KD on the RAS components in lung and heart tissues. Moreover, despite there being evidence that obesity enhances the expression of ACE2 and TMPRSS2 in lung tissue, to the best of our knowledge, no studies have evaluated whether a KD can affect molecular steps involved in SARS-CoV-2 infection. In this context, our study aims to compare the effects of different dietary interventions on the contents of viral entry proteins and RAS components in lung and heart tissues. Here, we provide a detailed analysis of how an obesogenic diet regulates ACE2 and TMPRSS2 contents, as well as AT_1_R and AT_2_R levels in lung and cardiac tissues. Additionally, we test whether a KD can alter the levels of these proteins and reduce the expression of inflammatory genes in the lungs.

## 2. Materials and Methods

### 2.1. Reagents

Protease (cOmplete Ultra Tablets) and phosphatase (PhosSTOP) inhibitors—were obtained from Roche Diagnostics GmbH (Mannheim, Germany). The Insulin ELISA kit was purchased from Millipore-Sigma (Burlington, MA, USA). The ACE1 (ab254222), ACE2 (ab239924), AT_1_R (ab124734), AT_2_R (ab92445) and TMPRSS2 (ab92323) antibodies were purchased from Abcam (Toronto, ON, Canada) and the β-actin (cat. no. 4967) antibody was purchased from Cell Signaling (Danvers, MA, USA).

### 2.2. Animals

Male albino rats from the Sprague-Dawley strain (Envigo, Indianapolis, IN, USA) weighing 200–250 g (initial weight)—were maintained in a constant-temperature (23 °C), with a fixed 12-h light/12-h dark cycle and fed for 16 weeks ad libitum either a standard chow diet (SC, 27.0%, 13.0%, and 60.0% of calories provided by protein, fat, and carbohydrates, respectively) a HF, sucrose-enriched (HFS) diet (20.0%, 60.0%, and 20.0% of calories provided by protein, fat, and carbohydrates [sucrose], respectively) or a KD (20%, 80% and 0% of calories provided by protein, fat and carbohydrates, respectively). The energy densities for the SC, HFS and ketogenic diets were 3.43, 5.24 and 6.14 kcal/g, respectively. The SC diet (standard rat chow, catalog # 5012) was purchased from TestDiet (Richmond, IN, USA). The HFS and ketogenic diets (catalog # D12492 and D03022101, respectively) were purchased from Research Diets Inc. (New Brunswick, NJ, USA). Energy intake was not significantly different between the three groups at weeks 0, 4 and 8 (Table 1). At week 16, the KD-fed animals had 17.9% and 18.1% lower energy intake than the SC and HFS-fed animals, respectively (Table 1).

### 2.3. Ethics Approval

The protocol containing all animal procedures described in this study was specifically approved by the Committee on the Ethics of Animal Experiments of York University (York University Animal Care Committee, YUACC, permit number: 2021-03) and performed strictly in accordance with the YUACC guidelines. All tissue extraction procedures were performed under ketamine/xylazine anaesthesia, and all efforts were made to minimize suffering [14]. All experiments in this study were carried out in compliance with the ARRIVE guidelines [15].

### 2.4. Glucose Monitoring and Determination of Plasma Insulin Concentrations

Blood from all animals in a fed state was collected between 15:00 and 16:00 h by saphenous vein bleeding and used to determine plasma glucose, using the OneTouch UltraMini blood glucose monitoring system from LifeScan Canada Ltd. (Vancouver, BC, Canada). Insulin was measured using a commercially available kit listed in the reagents section. All procedures were performed according to instructions provided by the manufacturer of the kit.

### 2.5. Western Blotting Analysis of ACE1, ACE2, AT_1_R, AT_2_R, and TMPRSS2 Protein Levels in Lung and Cardiac Tissues

Lung and heart tissues were homogenized in a buffer containing 25 mM Tris-HCl, 25 mM NaCl (pH 7.4), 1 mM MgCl_2_, 2.7 mM KCl, 1% Triton X-100 and protease and phosphatase inhibitors (Roche Diagnostics GmbH, Mannheim, Germany). Homogenates were centrifuged, the supernatant was collected, and an aliquot was used to measure protein by the Bradford method. Samples were diluted 1:1 (vol:vol) with 2× Laemmli sample buffer and heated to 95 °C for 5 min. Then, 25 μg of protein were loaded in each well. Samples were then subjected to SDS-PAGE, transferred to PVDF membrane, and probed for the proteins of interest. All primary antibodies were used at a dilution of 1:1000. All densitometry analyses were performed using the ImageJ program.

### 2.6. RNA Isolation and Quantitative PCR

Primers were designed using the software PrimerQuest (IDT) based on probe sequences available at the Affymetrix database (NetAffx™ Analysis Centre, http://www.affymetrix.com/analysis, accessed on 3 May 2020) for each given gene. RNA was isolated from lung and heart tissues using Trizol^TM^ (ThermoFisher Scientific, Waltham, MA, USA). Complimentary DNA (cDNA) was made from 2 μg of extracted RNA using the ABM OneScript cDNA Synthesis kit (Diamed, Mississauga, ON, Canada), according to the manufacturer’s instructions. Samples were run in duplicates on 96-well plates, and each 20 μL reaction contained 4 μL of cDNA, 0.4 μL of primer, 10 μL of Brightgreen 2× qPCR Mastermix (Diamed, Mississauga, ON, Canada) and 5.6 μL of RNase-free water. Real-time PCR analysis was performed using a Bio-Rad CFX96 Real Time PCR Detection System (Bio-Rad, Mississauga, ON, Canada) using the following amplification conditions: 95 °C (10 min); 40 cycles of 95 °C (15 s), 60 °C (60 s). All genes were normalized to the control gene β-actin, and values are expressed as fold increases relative to control [16]. Primer sequences utilized are shown in Table 2.

### 2.7. Statistical Analyses

Data were expressed as Mean ± SE. Statistical analyses were performed by using mixed-model analyses, and one-way ANOVAs and two way ANOVAs with Bonferroni multiple comparison post-hoc tests, as indicated in the figure legends. The GraphPad Prism software version 9.1.12 was used for all statistical analyses and for the preparation of all graphs. The level of significance was set to *p <* 0.05.

## 3. Results

*Body weight (BW), glycemia, and insulinemia*—HFS-fed animals gained significantly more weight than the SC and KD-fed animals (Table 3). This difference in BW was statistically significant at weeks 8 (409.77 ± 14.6 g in the HFS group vs. 370.21 ± 14.3 and 374.24 ± 6.6 g in the SC and KD groups, respectively), 12 (452.3 ± 15.7 g in the HFS group vs. 400.47 ± 15 and 404.69 ± 6.5 g in the SC and KD groups, respectively), and 16 (470.81 ± 17.1 g in the HFS group vs. 421.29 ± 15.7 and 425.54 ± 4.7 g in the SC and KD groups, respectively). With respect to blood glucose levels, there was no significant difference among the three groups at weeks 0 and 16 (Table 4). Insulinemia increased in both the HFS (1.90 ± 0.33 to 7.68 ± 1.54 ng/mL) and KD groups (2.18 ± 0.34 to 5.85 ± 0.66 ng/mL) from week 0 to week 16 (Table 4). However, at week 16, only the HFS had significantly higher blood insulin levels than the SC group (Table 4). In fact, the HFS diet-induced elevation in blood insulin concentration was 79%, relative to the SC animals (Table 4), which is an indication of insulin-resistance in these animals. In contrast, the SC and KD groups did not have statistically significantly different blood insulin levels at week 16 (Table 4).

### 3.1. ACE2 and TMPRSS2 Levels in Lung and Heart Tissues

**Figure 1:**
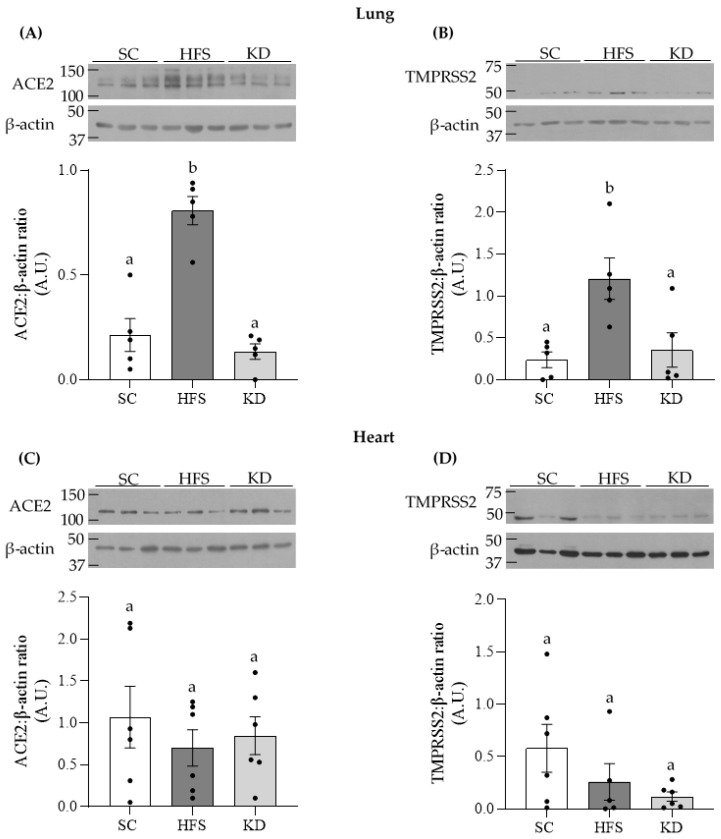
Lung contents of angiotensin converting enzyme 2 (ACE2) (**A**) and transmembrane protease serine 2 (TMPRSS2) (**B**) were significantly elevated by the HFS, but not by the KD. In contrast, diet did not alter the contents of these proteins in heart tissue (**C**,**D**). Statistical significance is denoted by different letters *p <* 0.05. Bars represent mean ± SEM. One-way ANOVA, *n* = 5–6. SC, standard chow; HFS, high-fat sucrose-enriched diet; KD, ketogenic diet.

We extracted lung and heart tissues from the animals for measurement of SARS-CoV-2 cellular entry proteins ACE2 and TMPRSS2. The HFS diet increased lung ACE2 content 3.8- and 6-fold, when compared to the SC and KD groups, respectively (Figure 1A). A similar pattern was observed for TMPRSS2 where, for the HFS diet, the levels of this protein increased by 5.1- and 3.4-fold, relative to the SC and KD groups, respectively (Figure 1B). However, in cardiac tissue, no significant differences were detected for ACE2 or TMPRSS2 levels when comparing the different dietary interventions (Figure 1C,D, respectively). These data indicate that the HFS diet increased SARS-CoV-2 entry proteins in lung tissue, but not in the heart.

### 3.2. ACE1, AT_1_R, and AT_2_R Levels in Lung and Heart Tissues

**Figure 2:**
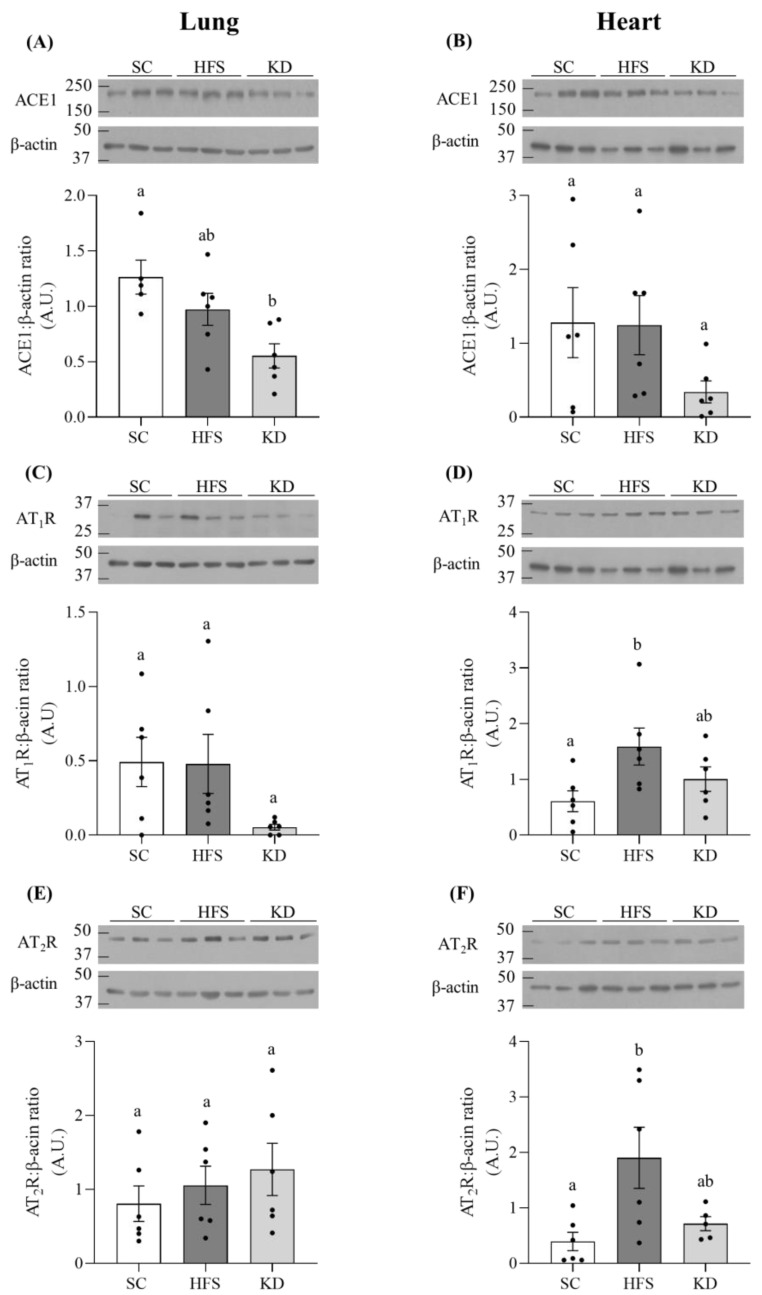
The KD attenuated angiotensin converting enzyme 1 (ACE1) and angiotensin II, type I receptor (AT_1_R) contents in pulmonary tissue (**A**,**C**, respectively), however only the former was found to be statistically significant. Angiotensin II, type 2 receptor (AT_2_R) was unaltered in the lungs, regardless of diet (**E**). On the other hand, dietary intervention did not influence heart ACE1 content (**B**), however AT_1_R and AT_2_R were significantly elevated in the cardiac tissues of HFS-fed rats (**D**,**F**, respectively). Significant differences are denoted by different letters *p <* 0.05. Bars represent mean ± SEM. One-way ANOVA, *n* = 5–6.

In pulmonary tissue, the KD attenuated ACE1 protein content by 56%, relative to the SC group (Figure 2A). Furthermore, KD also reduced AT_1_R protein content in this tissue; however, it was not statistically significant (Figure 2C). Pulmonary AT_2_R levels were not altered by diet either (Figure 2E). In cardiac tissue, ACE1 was not significantly different among the groups (Figure 2B). However, the HFS diet induced 2.6 and 4.9-fold increases in AT_1_R (Figure 2D) and AT_2_R (Figure 2F) levels, respectively, when compared to the SC group. The contents of these proteins were not significantly different between the SC- and KD-fed animals.

### 3.3. Comparison of ACE1, ACE2, AT_1_R, and AT_2_R in lung and Heart Tissues

**Figure 3:**
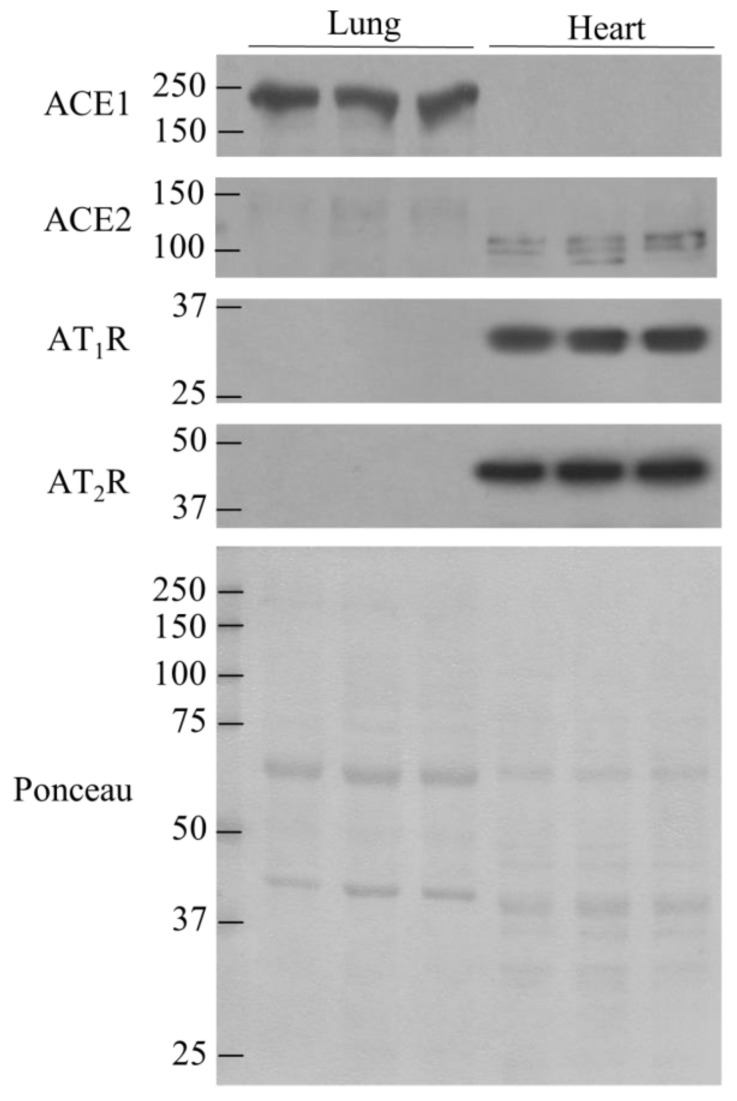
AT_1_R, AT_2_R and ACE2 are more abundant in heart tissue than in lung tissue. In contrast, lung tissue contains more ACE1. *n* = 3.

Upon observing the diet-induced changes in RAS proteins and the tissue-specific nature of these changes, we then decided to compare the contents of ACE1, ACE2, AT_1_R, and AT_2_R in the lungs and heart. Pulmonary tissue displayed higher levels of ACE1 than cardiac tissue (Figure 3), whereas more ACE2, AT_1_R, and AT_2_R was found in the heart than in the lungs. (Figure 3).

### 3.4. Gene Expressions of Mas1, tlr4, il6r, and tnfr1 in Lung and Heart Tissues

**Figure 4:**
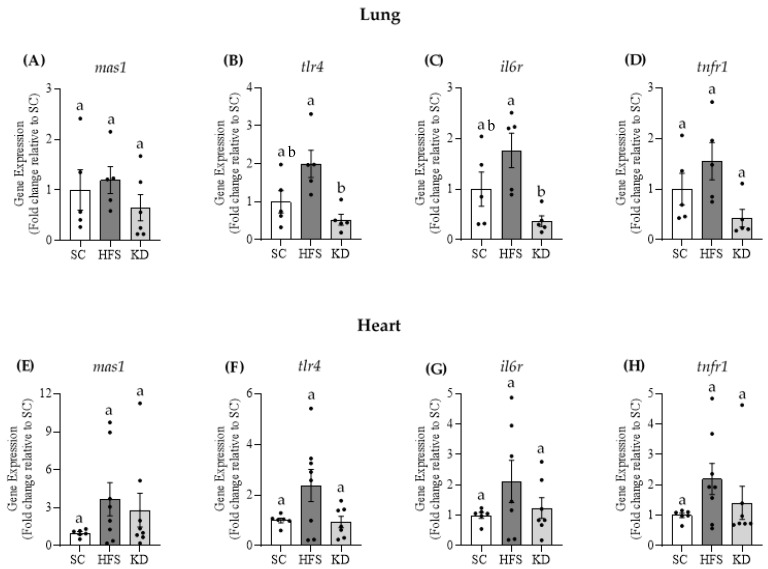
The KD does not affect *mas1* gene expression in lung (**A**) or heart (**E**) tissues. Interestingly, KD-feeding significantly reduces toll-like receptor 4 (*tlr4*) and interleukin-6 receptor (*il6r*) gene expression in pulmonary tissues (**B**,**C**, respectively), when compared to the HFS diet. The KD also reduced tumour necrosis factor receptor 1 (*tnfr1*) gene expression in lungs (**D**), though this was not statistically significant. *Tlr4, il6r and tnfr1* were not significantly altered by diet in cardiac tissues (**F**–**H**, respectively). Statistical significance is denoted by different letters *p <* 0.05. Bars represent mean ± SEM. One-way ANOVA, *n* = 5–8.

The gene expression of *mas1* was not significantly different among any of the three dietary interventions in either lung (Figure 4A) or heart tissue (Figure 4E). However, the gene expressions of *tlr4* and *il6r* were significantly reduced by 74% and 79%, respectively, in the lungs of KD-fed animals, when compared to the HFS animals (Figure 4B,C, respectively). KD also suppressed *tnfr1* gene expression in this tissue by ~73% (Figure 4D), although this was not statistically significant (*p* = 0.059). In the heart, no significant differences were found for *tlr4*, *il6r*, and *tnfr1* gene expressions among the three diet groups (Figure 4F–H, respectively). Altogether, these results indicate that the KD exerted an anti-inflammatory effect in the lungs, but not in the heart.

## 4. Discussion

Here, we show that obesogenic and ketogenic diets regulate in a distinct and tissue-specific manner proteins of the RAS, as well as major components of the molecular machinery involved in cellular infection by SARS-CoV-2. In fact, rats fed the HFS obesogenic diet displayed elevated insulinemia and much higher levels of ACE2 and TMPRSS2 in the lungs than animals fed either SC or a KD. This is consistent with previous findings of increased lung levels of ACE2 and TMPRSS2 in diabetic mice [7], and with reports linking insulin resistance and Type 2 diabetes to severe COVID outcomes [2,7]. However, to our knowledge, this is the first study to show that besides attenuating weight gain and insulinemia, a KD maintained lower ACE2 and TMPRSS2 protein levels in the lungs in comparison to a HFS diet. This is relevant for the prevention of severe COVID-19-related illness, given that ACE2 and TMPRSS2 have been identified as crucial components of the molecular machinery used by SARS-CoV-2 to infects cells [5]. In fact, use of the specific TMPRSS2 inhibitor, camostat mesylate, has been shown to inhibit SARS-CoV-2 entry in Caco-2 cells, and anti-ACE2 antibody incubation with Vero cells blocked SARS-CoV-1 and SARS-CoV-2 entry [5]. Thus, the observed elevation in these two proteins in the lungs of HFS-fed rats provides a plausible mechanism whereby obese individuals are more susceptible to severe infection with SARS-CoV-2. The underlying mechanisms that explain this diet-induced elevation in ACE2 have not been fully elucidated [17]. However, under conditions of obesity, adipose tissue secretes angiotensinogen, which can eventually be converted to Ang-II, leading to hyperactivation of the ACE1/Ang-II/AT_1_R arm of the RAS and subsequent tissue damage [2,8]. Hyperinsulinemia may also cause RAS dysregulation [8]. In this context, the observed elevation in ACE2 is likely a counterregulatory mechanism to attenuate the detrimental effects induced by obesity-mediated RAS hyperactivation [17]. Furthermore, non-alcoholic fatty liver disease (NAFLD) is a major comorbidity of obesity, and it has been linked to increased hepatic expression of ACE2 and TMPRSS2 [18], which could contribute to the aggravate morbidity in SARS-CoV-2 patients. Importantly, ketogenic diets have been shown to be very effective in combating NAFLD [19,20,21] and attenuate the potential deleterious effects of SARS-CoV-2 in the liver. However, we did not assess any variable associated with NAFLD in our studies; therefore, we could not make any inferences about hepatic expression of SARS-CoV-2 critical entry points.

Although there were no significant alterations in ACE2 and TMPRSS2 in the heart with any of the dietary interventions, ACE2 was found to be more abundant in cardiac than in lung tissue. Thus, because the obesogenic diet significantly increased ACE2 in the lungs, COVID-19 infection still poses a significant risk for cardiac health in obese individuals, since it is one of the primary points of infection. This could potentially enhance the severity of the infection in pulmonary tissues, leading to viral spill over, whereby the virus can access other tissues, including the heart, which is already abundant in ACE2 and, as a result, predisposed to infection and damage [2,6]. The roles of ACE2 in SARS-CoV-2 infection, as well as the RAS, are not independent. Thus, the other RAS components are also emerging as therapeutic targets to treat illness related to COVID-19. In fact, the use of angiotensin receptor blockers (ARB) or ACE inhibitors have been proposed as treatments for SARS-CoV-2 patients [2]. This serves to increase flux through the ACE2/Ang(1,7)/MasR and AT_2_R arm of the RAS and to promote anti-inflammatory mechanisms that counteract the COVID-induced cytokine storm [2,6,8]. In the present study, we observed no alteration in AT_1_R or AT_2_R in lung tissues with any of the three dietary interventions. However, the KD did significantly reduce ACE1 levels. This indicates that the RAS system was shifted away from the ACE1/Ang-II/AT_1_R arm and towards the counterregulatory, anti-inflammatory RAS arm, given that we also observed reductions in *tlr4,* and *il6r* gene expressions, as well as a trend to decrease *tnfr1* gene expression. The observed reduction in the expression of these inflammatory genes is relevant, as IL-6 and TNFα are two major cytokines that facilitate the coronavirus-induced cytokine storm [2]. Furthermore, Zhao et al. demonstrated that TLR4 inhibition suppressed interleukin-1β release in cells treated with the SARS-CoV-2 S protein [22].

Our findings are in line with other studies that report the anti-inflammatory nature of the KD [13,23], and suggest that this diet could reduce the risk of severe infection and inflammatory response related to COVID-19. We have not investigated the mechanism by which the KD led to lower levels of expression of inflammatory genes in comparison to the obesogenic diet. However, the KD is characterized by increased liver production of ketones [24]. Thus, the anti-inflammatory effect of the KD could be at least partially attributed to increased levels of circulating ketones, particularly β-hydroxybutyrate (β-HB), upon KD feeding [25]. In fact, β-HB has been demonstrated to block the nucleotide-binding domain leucine-rich-containing family pyrin domain-containing-3 (NLRP3) inflammasome and NLRP3 inflammasome-mediated interleukin (IL)-1β and IL-18 production by monocytes [26]. Additionally, the KD has been shown to exert its anti-inflammatory effects by inhibiting nuclear factor kappa-light-chain-enhancer of activated B cells (NF-kB) activation, suppressing the activity of histone deacetylases (HDACs), reducing the production of reactive oxygen species (ROS), and by improving mitochondrial respiration [24]. Thus, all these KD effects can ameliorate chronic low-grade inflammation and its associated diseases. It is important to mention that the KD used in this study was completely devoid of carbohydrates, which differs from the commonly used KD that recommends low carbohydrate content (less than 50 g/day) and not the complete elimination of this macronutrient from the diet [27]. Thus, caution should be taken when extrapolating our findings to human subjects. However, the absence of carbohydrates in the diet allowed us to identify that it is the combination of HF with sucrose, as opposed to HF alone, that promotes the deleterious metabolic effects associated with increased levels of SARS-CoV-2 entry proteins and inflammation in lung and heart tissues in obesity.

We did not find any significant difference in the cardiac gene expressions of Mas1 or the aforementioned inflammatory genes, although a trend to increase the latter in the HFS-fed rats was detected. However, the HFS diet did increase AT_1_R content, which was also met by a similar increase in AT_2_R. This likely served to offset the proinflammatory response mediated by AT_1_R. Indeed, it has been reported that AT_2_R increases in response to AT_1_R hyperactivation and injury [2,11]. Interestingly, AT_1_R and AT_2_R are more abundant in the heart than in the lungs. In this context, it is possible that the elevated expression of these receptors allows for an enhanced flexibility of this tissue to regulate its inflammatory status. It is important to note that AT_1_R and AT_2_R levels did not change in the hearts of KD-fed animals, relative to the SC animals, suggesting that this diet did not provoke RAS activation. Unlike the lungs, heart ACE1 content was not altered by diet. This could be attributed to the lower ACE1 content in the heart, compared to the lungs, suggesting that the reliance of the former on ACE1 for RAS regulation is lower than the latter, where there was a diet-induced alteration in the content of this protein. This is consistent with the notion that the ACE1-dependent conversion of Ang-I to Ang-II takes place predominantly in pulmonary circulation [10].

In conclusion, these data indicate that the HFS diet increases body weight, insulinemia RAS hyperactivation and lung ACE2 and TMPRSS2, which could explain the predisposition of obese individuals to severe COVID-19 outcomes. In contrast, the KD did not elicit the same elevations in weight gain and insulinemia as the HFS diet did. Our data also suggests that there is not a hyperactivation of the RAS in KD-fed animals, as indicated by the reductions in pulmonary ACE1 and the expressions of inflammatory genes, as well as unaltered cardiac AT_1_R and AT_2_R contents. Thus, a counterregulatory elevation in lung ACE2 was not observed in KD animals. Unlike the lungs, the heart does not experience a diet-induced alteration in ACE2, however the abundance of this protein, relative to the lungs, makes it a susceptible organ to SARS-CoV-2 damage, following viral spill over from pulmonary tissues. In this context, despite altering RAS proteins in a tissue-specific manner, it is clear that the KD offers a promising potential therapeutic tool for its reduction in SARS-CoV-2 entry proteins and its induction of an anti-inflammatory profile.

## Figures and Tables

**Table 1:** Energy intake at weeks 0, 4, 8, and 16 in the SC-, HFS-, and KD-fed animals.

	Week	SC	HFS	KD
Energy intake (kcal/rat/day)	0	69.23 ± 3.8	66.49 ± 2.3	66.49 ± 2.9
	4	72.60 ± 4.2	68.37 ± 4.0	63.83 ± 0.76
	8	65.83 ± 2.9	66.94 ± 3.2	59.82 ± 1.1
	16	69.65 ± 2.1	69.75 ± 6.0	57.15 ± 5.0 *

* *p <* 0.05 vs. SC and HFS within the respective week. Data are expressed as mean ± SEM. Two-way ANOVA. *n* = 3–7. SC, standard chow; HFS, high-fat sucrose-enriched diet; KD, ketogenic diet.

**Table 2:** Primer sequences for qPCR.

	Forward	Reverse
mas1	5′-CGCCAACCCTTTCATCTACT-3′	5′-CCTAGGTTGCATCTCGTCTTT-3′
tlr4	5′-ACCTAAGGAGAGGAGGCTAAG-3′	5′-GGTAACTGCAGCACACTACA-3′
il6r	5′-TGGAGCAGACAGAGAGACTT-3′	5′-AGCTTACAGGTAACAGAGCATAAA-3′
tnfr1	5′-CCCTGTGAACCTCCTCTTTG-3′	5′-CTATGTACACCAAGTCGGTAGC-3′
β-actin	5′-GTGAAAAGATGACCCAGATC-3′	5′-CACCGCCTGGATGGCTACGT-3′

**Table 3:** Body weight of SC-, HFS-, and KD-fed rats at the beginning of the study (week 0) and after 4, 8, 12, and 16 weeks of dietary intervention.

		Duration of Study (Weeks)				
	Groups	0	4	8	12	16
Body mass (g)	SC	231.97 ± 7.1	317.21 ± 10.4	370.21 ± 14.3	400.47 ± 15.0	421.29 ± 15.7
	HFS	236.18 ± 5.4	345.97 ± 10.7	409.77 ± 14.6 *	452.3 ± 15.7 *	470.81 ± 17.1 *
	KD	230.12 ± 3.6	321.21 ± 4.5	374.24 ± 6.6	404.69 ± 6.5	425.54 ± 4.7

* *p <* 0.05 vs. SC and KD within the respective weeks. Data are expressed as mean ± SEM. Mixed-effects model analysis. *n* = 7–9.

**Table 4:** Glycemia and insulinemia levels at the beginning (week 0) and at the end of the dietary intervention (week 16) in SC-, HFS-, and KD-fed rats.

		Duration of Study (Weeks)	
Blood Parameters	Groups	0	16
Glucose (mmol/L)	SC	6.85 ± 0.12	5.72 ± 0.18 ^#^
	HFS	6.77 ± 0.21	5.91 ± 0.10 ^#^
	KD	6.59 ± 0.12	5.71 ± 0.07 ^#^
Insulin (ng/mL)	SC	2.16 ± 0.50	4.30 ± 0.43
	HFS	1.90 ± 0.33	7.68 ± 1.54 *^#^
	KD	2.18 ± 0.34	5.85 ± 0.66 ^#^

* *p <* 0.05 vs. SC within the respective week; # *p <* 0.05 vs. week 0 measurement. Data are expressed as mean ± SEM. Mixed-effects model analysis, *n* = 6–7.

## Data Availability

All data are reported in the manuscript.
